# Supplementary material for: Characteristics of COVID-19 Patients Admitted to Intensive Care Unit in Multispecialty Hospital of Riyadh, Saudi Arabia: A Retrospective Study
Source: Healthcare (Basel). 2023 Sep 8;11(18):2500. doi: 10.3390/healthcare11182500 (PMC10530388; doi:10.3390/healthcare11182500)
Supplement: Supplementary file 1 [file healthcare-11-02500-s001.zip › healthcare-2566856-supplementary.pdf]

Article

Characteristics of COVID-19 Patients Admitted to Intensive Care Unit in Multispecialty Hospital of Riyadh, Saudi Arabia: A retrospective study

Mansour Almuqbil[1*], Ali Ibrahim Almoteer[2], Alwaleed Mohammed Suwayyid[2], Abdulaziz Hussain Bakarman[2], Raed Fawaz alrashed[2], Majed Alrobish[2], Fahad Alasalb[2], Abdulaziz Abdulrahman Alhusaynan[2], Mohammed Hadi Alnefaie[2], Abdullah Saud Altayar[2], Saad Ebrahim Alobid[3], Moneer E. Almadani[4], Ahmed Alshehri[5], Adel Alghamdi[6], Syed Mohammed Basheeruddin Asdaq[7*]

[1]Department of Clinical Pharmacy, College of Pharmacy, King Saud University, Riyadh 11451, Saudi Arabia, mmetwazi@ksu.edu.sa (M.A.)

[2]Department of Pharmacy Practice, College of Pharmacy, AlMaarefa University, Dariyah, 13713, Riyadh, Saudi Arabia, sasdaq@gmail.com (S.M.B.A); Aalmotair@ksu.edu.sa (A.I.A); Dr.alwaleedswayyid@gmail.com (A.M.S); Abdulazizh148@gmail.com (A.H.B); Raed.fa95@gmail.com (R.F.A); 171120154@student.mcst.edu.sa (M.A); 161120124@student.mcst.edu.sa (F.A); Phd.az95@gmail.com (A.A.A); bn.hadi@gmail.com (M. H.A); oooatooo@hotmail.com (A.S.A).

[3]Department of Pharmacology and Toxicology, College of Pharmacy, King Saud University, Riyadh, Saudi Arabia. saaalobaid@ksu.edu.sa

[4]Department of Clinical Medicine, College of Medicine, AlMaarefa University, Dariyah, 13713, Riyadh, Saudi Arabia, mmadani@mcst.edu.sa

[5]Department of Pharmacology, College of Clinical Pharmacy, Imam Abdulrahman Bin Faisal University, King Faisal Road, Dammam 31441, Saudi Arabia, adalshehri@iau.edu.sa

[6]Department of Pharmaceutical Chemistry, Faculty of Clinical Pharmacy, Al Baha University, P.O. Box 1988 Al Baha, Saudi Arabia, Ai.alghamdi@bu.edu.sa

[7]Department of Pharmacy Practice, College of Pharmacy, AlMaarefa University, Dariyah, 13713, Riyadh, Saudi
Arabia, sasdaq@gmail.com (SMBA),

* Correspondence: sasdaq@gmail.com/sasdag@mcst.edu.sa (SMBA); mmetwazi@ksu.edu.sa (MA)

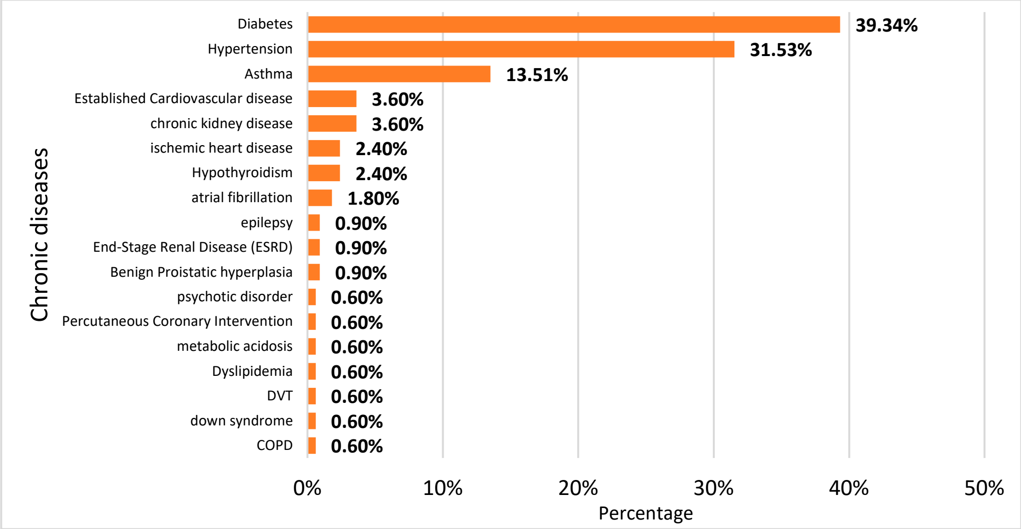

Supplementary Figure S1. Percentage distribution of chronic disease.

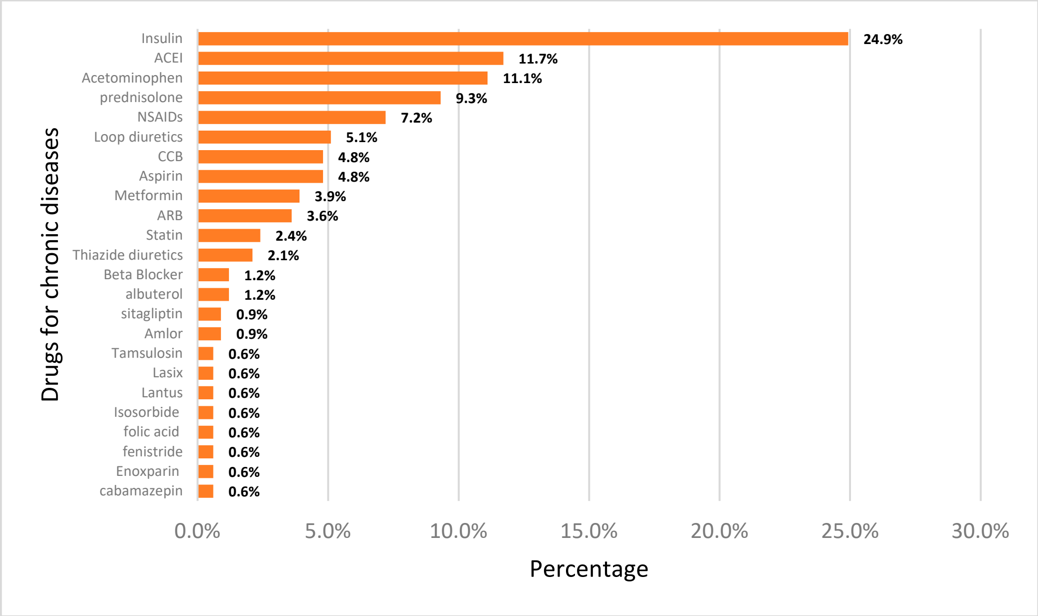

Supplementary Figure S2. Percentage distribution of drugs used for chronic diseases.

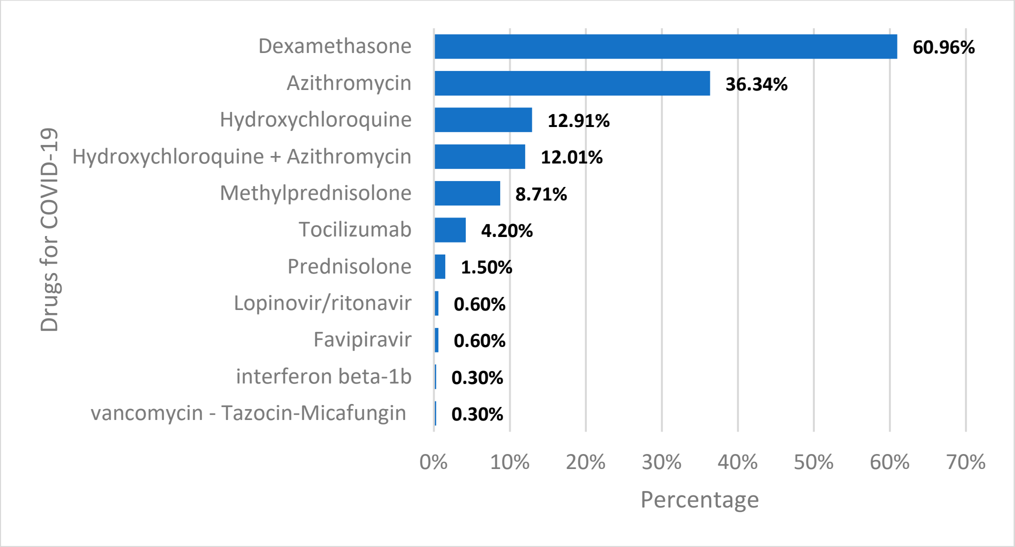

Supplementary Figure S3. Percentage distribution of drugs used for COVID-19.
